# Protocol to analyze dysregulation of the eIF4F complex in human cancers using R software and large public datasets

**DOI:** 10.1016/j.xpro.2022.101880

**Published:** 2022-12-12

**Authors:** Su Wu, Gerhard Wagner

**Affiliations:** 1 Department of Biological Chemistry and Molecular Pharmacology, Harvard Medical School, Boston, MA 02115, USA

**Keywords:** Bioinformatics, Cancer, RNAseq, Proteomics

## Abstract

Understanding dysregulation of the eukaryotic initiation factor 4F (eIF4F) complex across tumor types is critical to cancer treatment development. We present a protocol and accompanying R package “eIF4F.analysis”. We describe analysis of copy number status, gene abundance and stoichiometry, survival probability, expression covariation, correlating genes, mRNA/protein correlation, and protein co-expression. Using publicly available large multi-omics data, eIF4F.analysis permits computationally derived and statistically powerful inferences regarding initiation factor regulation in human cancers and clinical relevance of protein interactions within the eIF4F complex.

For complete details on the use and execution of this protocol, please refer to Wu and Wagner (2021).^1^

## Highlights

• An R package to analyze eIF4F dysregulation, using large multi-omics datasets
• Detailed steps for software installation, data download, and library initialization
• Guidelines for deriving biological and clinical inferences from multiple analyses
• Illustrated code structure explains bioinformatics pipeline assembly technique

## Abstract (editor highlights)

Publisher’s note: Undertaking any experimental protocol requires adherence to local institutional guidelines for laboratory safety and ethics.

## Graphical abstract

**Figure:**
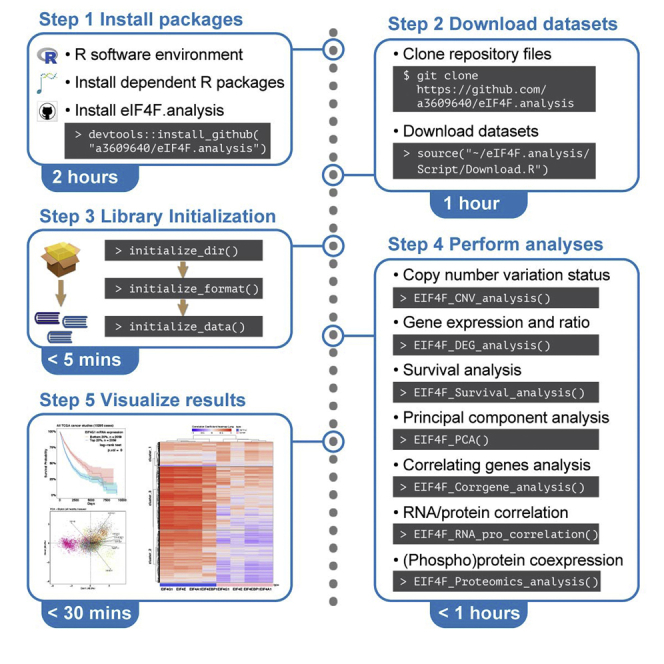

## Before you begin

### Overview

In complex biological systems, biological processes rely on participation of multiple proteins through their physical and specific functional interactions. Clinical relevance of protein-protein interactions (PPIs) has been traditionally investigated through wet-lab approaches using tissue cultures and animal models, which are often complicated by easily-perturbed cell culture conditions or poor recapitulation of human diseases.^2^ To mitigate these complications, we developed a computational method to infer PPIs for eukaryotic translation initiation complexes and their clinical relevance in cancers from human data. We produced an R package that employs a series of computational analyses including copy number comparison, survival regression modeling, quantification of subunit abundance and stoichiometry, differential gene expression with unsupervised machine learning, differential gene correlation and pathway activity, and mRNA/protein correlations.

The validity of our method derives from the biological requirement for interacting proteins to be simultaneously present within a cell, which implies that synthesis and degradation of interacting proteins ought also to coincide.^3^ The statistical association between *in vivo* gene co-expression and protein interactions has been reported after analyzing large-scale data from multiple species including human.^4^^,^^5^^,^^6^ When using expression data from clinical samples of The Cancer Genome Atlas (TCGA), Genotype-Tissue Expression (GTEx), Cancer Cell Line Encyclopedia (CCLE), and Clinical Proteomic Tumor Analysis Consortium (CPTAC) databases, our method allows to assess functional and clinical relevance of PPIs by associating protein co-expression with malignant phenotype or disease progression. Because our approach leverages large sample groups, it benefits from high statistical power.

**Figure 1:**
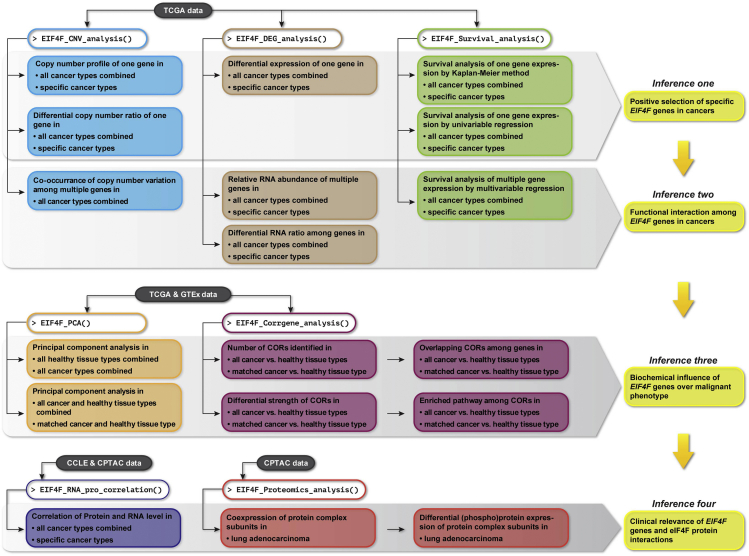
Summary of outputs from all functions and biological inferences

As we previously published,^1^ we have applied this approach to assess PPIs of eIF4F subunits in healthy and cancerous tissues. It yielded high-value, cost-efficient biochemical insights into functional and regulatory effects driven by eIF4F subunit interactions across tumor types, in addition to those previously published from isolated cell lines cultivated in wet labs. Figure 1 summarizes the major analyses accomplished by functions in eIF4F.analysis package, and a step-by-step guide to infer the clinical relevance of *EIF4F* genes and their protein interactions from analysis results. Our innovative computational method can be extended for broader application to reveal the clinical relevance of PPIs in assorted disease conditions, given additional disease-related datasets, custom lists of candidate protein complexes with subunits, and implementation of more association analyses. However, the protocol presented here is narrowly targeted to the production of our eIF4F results.

### Preparation one: Installing RStudio/R

eIF4F.analysis package was developed in RStudio and implemented in the R programming language.

1. Download & install R 4.2.1, if not already installed.
2. Download & install RStudio, if not already installed: (https://www.rstudio.com/products/rstudio/download/).

### Preparation two: Installing dependent R packages

The required packages will be automatically installed the first time you run eIF4F.analysis.

3. The following commands are useful to install dependent R packages manually in RStudio.

# use Bioconductor version 3.15 for package installation

> if (!require("BiocManager", quietly = TRUE))

install.packages("BiocManager")

> BiocManager::install(version = "3.15")

# install required R packages:

> bio_pkgs <- c("AnnotationDbi", "circlize", "clusterProfiler",

  "ComplexHeatmap", "corrplot", "data.table",

  "devtools", "dplyr", "EnvStats”, "eulerr",

  "factoextra", "FactoMineR", "forcats", "forestplot",

  "ggfortify","ggplot2", "ggpubr", "graphics",

  "grDevices", "grid", "limma", "log4r", "missMDA",

  "org.Hs.eg.db", "purrr", "R.utils", "RColorBrewer",

  "ReactomePA", "RCurl","readr", "readxl", "reshape2",

  "rlang", "scales", "stats", "stringr", "survival",

  "survivalAnalysis", "tibble", "tidyr", "tidyselect",

  "utils")

> BiocManager::install(bio_pkgs)

# load required packages

> lapply(bio_pkgs, require, character.only = TRUE)

***Note:*** Except for the "RCurl," "R.utils," and "utils" packages that are required for Download.R file, all dependent packages will be installed in the next step. However, installation of all dependent package takes a long time and sometimes gives installation errors related to the setting of individual users. Thus, we recommend users to manually install dependent packages before installing eIF4F.analysis package.

**CRITICAL:** If installation of some dependent packages requires to install or update non-Bioconductor packages, use install.packages() for non-Bioconductor packages.

### Preparation three: Installing and loading eIF4F.analysis

Install the development version of eIF4F.analysis package from GitHub with the following commands:

4. Install eIF4F.analysis package in RStudio.

> devtools::install_github("a3609640/eIF4F.analysis")

5. Load eIF4F.analysis package in RStudio.

> library(eIF4F.analysis)

### Preparation four: Clone the repository files

We provide two R scripts in our GitHub repository for users to download datasets and to run eIF4F.analysis package. Users need to clone the package files from GitHub, and set up the directories for input and output files.

**Figure 2:**
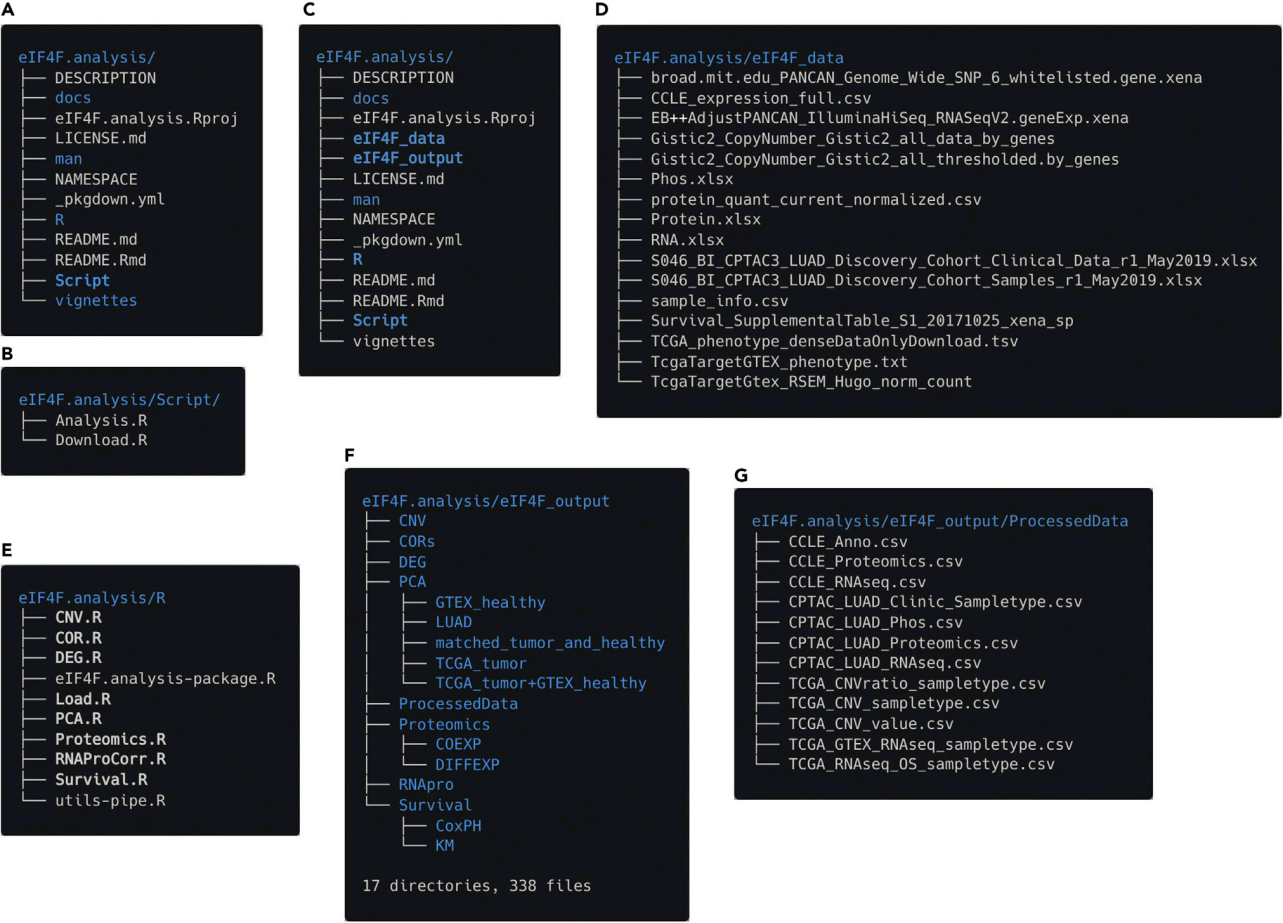
Terminal outputs for the preparation and analysis steps (A) The files downloaded under the eIF4F.analysis folder after the GitHub repository cloning. (B) Two R scripts for users to acquire datasets and perform analyses, stored under the eIF4F.analysis/Script folder. (C) The data and output folders added under the eIF4F.analysis folder after execution of the Download.R script. (D) The data files downloaded under the eIF4F.analysis/eIF4F_data folder after execution of the Download.R script. (E) The R scripts for analysis functions, stored under the eIF4F.analysis/R folder. (F) The subdirectories generated under the eIF4F.analysis/eIF4F_output folder after execution of the initialize_dir() command. (G) The csv files generated under the eIF4F.analysis/eIF4F_output/ProcessedData folder after execution of the initialize_data() command.

6. Open the terminal and run the following command line to clone our GitHub repository for eIF4F.analysis package. This step will store all package files under home directory as ∼/eIF4F.analysis (Figure 2A).

$ git clonehttps://github.com/a3609640/eIF4F.analysis

***Note:*** Installing eIF4F.analysis package in the Linux system is highly recommended.

7. Under the ∼/eIF4F.analysis/Script folder (Figure 2B), there are two R scripts Download.R and Analysis.R for users to acquire datasets and perform analyses. Open Download.R file in RStudio, verify the directories for input and output files as following.

# default directory for data download and output storage

> data_file_directory <- Sys.getenv(c("DATA_FILE_DIRECTORY"), unset = "∼/eIF4F.analysis/eIF4F_data")

> output_directory <- Sys.getenv(c("OUTPUT_DIRECTORY"), unset = "∼/eIF4F.analysis/eIF4F_output")

**CRITICAL:** By default, Download.R generates root directory paths ∼/eIF4F.analysis/eIF4F_data and ∼/eIF4F.analysis/eIF4F_output. These two directory paths are also set inside the package (in Load.R), and the values must agree in both places. The descriptions of the steps that follow assume the use of the default eIF4F_output location.

### Preparation five: Download datasets

**Timing: 1 h**

Execution of Download.R script creates two folders: ∼/eIF4F.analysis/eIF4F_data and ∼/eIF4F.analysis/eIF4F_output (Figure 2C). Download.R then downloads all needed datasets (TGCA, GTEx, CPTAC, CCLE, etc.) from URLs to ∼/eIF4F.analysis/eIF4F_data and decompresses them. After the completion of the download and unzip steps, the eIF4F_data folder contains 16 data files (Figure 2D), with a collective size of 15 GB. ∼/eIF4F.analysis/eIF4F_output is to store all the analyzed results in the following steps.

8. Run the Download.R file in RStudio with the following command line.

> source("∼/eIF4F.analysis/Script/Download.R")

***Note:*** Download times were observed with a 100 Mbps internet connection. Remote data sources mentioned here may offer limited and lower bandwidth.

**CRITICAL:** By default, user will be able to clone all the scripts and R codes from Github repository, to download datasets, and to store analysis results (by default) under a single folder ∼/eIF4F.analysis.

## Key resources table

**Table:** 

REAGENT or RESOURCE	SOURCE	IDENTIFIER
**Software and algorithms**		

R (version 4.2.1)	Comprehensive R Archive Network (CRAN)	https://www.r-project.org
RStudio (2022.07.1 build 554)	RStudio: Integrated Development Environment for R	http://www.rstudio.com/
BiocManager (version 3.15)	Bioconductor (3.15)	https://www.bioconductor.org/install/
AnnotationDbi (version 1.58.0)	Bioconductor (3.15)	https://bioconductor.org/packages/release/bioc/html/AnnotationDbi.html
circlize (version 0.4.15)	CRAN	https://cran.r-project.org/web/packages/circlize/index.html
clusterProfiler (version 4.4.4)	Bioconductor (3.15)	https://bioconductor.org/packages/release/bioc/html/clusterProfiler.html
ComplexHeatmap (version 2.12.1)	Bioconductor (3.15)	https://www.bioconductor.org/packages/release/bioc/html/ComplexHeatmap.html
corrplot (version 0.92)	CRAN	https://cran.r-project.org/web/packages/corrplot/
data.table (version 1.14.2)	CRAN	https://cran.r-project.org/web/packages/data.table/index.html
devtools (version 2.4.4)	CRAN	https://cran.r-project.org/web/packages/devtools/index.html
dplyr (version 1.0.10)	CRAN	https://cran.r-project.org/web/packages/dplyr/
eIF4F.analysis (version 1.0.0)	This article	https://doi.org/10.5281/zenodo.7226224
EnvStats (version 2.7.0)	CRAN	https://cran.r-project.org/web/packages/EnvStats/index.html
factoextra (version 1.0.7)	CRAN	https://cran.r-project.org/web/packages/factoextra/index.html
FactoMineR (version 2.6)	CRAN	https://cran.r-project.org/web/packages/FactoMineR/index.html
forcats (version 0.5.2)	CRAN	https://cran.r-project.org/web/packages/forcats/index.html
forestplot (version 3.0.0)	CRAN	https://cran.r-project.org/web/packages/forestplot/index.html
ggfortify (version 0.4.14)	CRAN	https://cran.r-project.org/web/packages/ggfortify/index.html
ggplot2 (version 3.3.6)	CRAN	https://cran.r-project.org/web/packages/ggplot2/index.html
ggpubr (version 0.4.0)	CRAN	https://cran.r-project.org/web/packages/ggpubr/index.html
graphics (version 4.2.1)	CRAN	https://stat.ethz.ch/R-manual/R-devel/library/graphics/html/graphics-package.html
grDevices (version 4.2.1)	CRAN	https://stat.ethz.ch/R-manual/R-devel/library/grDevices/html/grDevices-package.html
grid (version 4.2.1)	CRAN	https://stat.ethz.ch/R-manual/R-devel/library/grid/html/grid-package.html
limma (version 3.52.3)	Bioconductor (3.15)	https://bioconductor.org/packages/release/bioc/html/limma.html
log4r (version 0.4.2)	CRAN	https://cran.r-project.org/web/packages/log4r/index.html
magrittr (version 2.0.3)	CRAN	https://cran.r-project.org/web/packages/magrittr/index.html
missMDA (version 1.18)	CRAN	https://cran.r-project.org/web/packages/missMDA/index.html
org.Hs.eg.db (version 3.15.0)	Bioconductor (3.15)	https://bioconductor.org/packages/release/data/annotation/html/org.Hs.eg.db.html
purrr (version 0.3.4)	CRAN	https://cran.r-project.org/web/packages/purrr/index.html
R.utils (version 2.12.0)	CRAN	https://cran.r-project.org/web/packages/R.utils/index.html
RColorBrewer (version 1.1-3)	CRAN	https://cran.r-project.org/web/packages/RColorBrewer/
RCurl (version 1.98-1.9)	CRAN	https://cran.r-project.org/web/packages/RCurl/index.html
ReactomePA (version 1.40.0)	CRAN	https://bioconductor.org/packages/release/bioc/html/ReactomePA.html
readr (version 2.1.3)	CRAN	https://cran.r-project.org/web/packages/readr/index.html
readxl (version 1.4.1)	CRAN	https://cran.r-project.org/web/packages/readxl/
reshape2 (version 1.4.4)	CRAN	https://cran.r-project.org/web/packages/reshape2/index.html
rlang (version 1.0.6)	CRAN	https://cran.r-project.org/web/packages/rlang/index.html
scales (version 1.2.1)	CRAN	https://cran.r-project.org/web/packages/scales/
stats (version 4.3.0)	CRAN	https://stat.ethz.ch/R-manual/R-devel/library/stats/html/stats-package.html
stringr (version 1.4.1)	CRAN	https://cran.r-project.org/web/packages/stringr/
survival (version 3.4-0)	CRAN	https://cran.r-project.org/web/packages/survival/index.html
survivalAnalysis (version 0.3.0)	CRAN	https://cran.r-project.org/web/packages/survivalAnalysis/index.html
tibble (version 3.1.8)	CRAN	https://cran.r-project.org/web/packages/tibble/index.html
tidyr (version 1.2.1)	CRAN	https://cran.r-project.org/web/packages/tidyr/index.html
tidyselect (version 1.1.2)	CRAN	https://cran.r-project.org/web/packages/tidyselect/index.html

**Deposited data**		

TCGA CNV dataset (thresholded)	UCSC Xena	Gistic2_CopyNumber_Gistic2_all_thresholded.by_genes
TCGA CNV dataset	UCSC Xena	Gistic2_CopyNumber_Gistic2_all_data_by_genes
TCGA CNV ratio dataset	UCSC Xena	broad.mit.edu_PANCAN_Genome_Wide_SNP_6_whitelisted.gene.xena
TCGA RNA-Seq dataset	UCSC Xena	EB++AdjustPANCAN_IlluminaHiSeq_RNASeqV2.geneExp.xena
TCGA sample type annotation	UCSC Xena	TCGA_phenotype_denseDataOnlyDownload.tsv
TCGA overall survival data	UCSC Xena	Survival_SupplementalTable_S1_20171025_xena_sp
TCGA and GTEx RNA-Seq dataset	UCSC Xena	TcgaTargetGtex_RSEM_Hugo_norm_count
TCGA and GTEx sample type annotation	UCSC Xena	TcgaTargetGTEX_phenotype.txt
CPTAC LUAD Sample Annotation	CPTAC LUAD Discovery Study - Proteome	PDC Study Identifier: PDC000153 S046_BI_CPTAC3_LUAD_Discovery_Cohort_Samples_r1_May2019.xlsx
CPTAC LUAD Clinical Data	CPTAC LUAD Discovery Study - Proteome	PDC Study Identifier: PDC000153 S046_BI_CPTAC3_LUAD_Discovery_Cohort_Clinical_Data_r1_May2019.xlsx
CPTAC LUAD RNA-Seq data	Gillette et al. (2020)^7^	https://www.cell.com/cms/10.1016/j.cell.2020.06.013/attachment/ab6fcc71-22e2-4ce9-9947-926fba367bd6/mmc2.xlsx File name: RNA.xlsx
CPTAC LUAD proteomics data	Gillette et al. (2020)^7^	https://www.cell.com/cms/10.1016/j.cell.2020.06.013/attachment/49a46b71-468b-45d1-826a-721fa734eff0/mmc3.xlsx File name: Protein.xlsx
CPTAC LUAD phosproteomics data	Gillette et al. (2020)^7^	https://www.cell.com/cms/10.1016/j.cell.2020.06.013/attachment/49a46b71-468b-45d1-826a-721fa734eff0/mmc3.xlsx File name: Phos.xlsx
CCLE RNA-Seq data	Broad	CCLE_expression_fill.csv
CCLE proteomics data	Nusinow et al. (2020)^8^	protein_quant_current_normalized.csv
CCLE annotation data	Broad	sample_info.csv

**Other**		

Intel i7-8700k CPU, 64 GB RAM (DDR4-3000, non-ECC), Samsung NVMe Pro SSD, Pop!_OS 22.04 LTS	N/A	N/A

## Materials and equipment

• Software: R version 4.2.1 and RStudio (2022.07.1 build 554).
• Operating systems: The package was developed and tested with Linux OS (Pop!_OS 22.04 LTS).

**CRITICAL:** We recommend Linux to run the package.

• Computer hardware:
  ○ Memory: 48 GB minimum.
  ○ Processors: tested with Intel Core i7-8700k CPU (6 cores), Intel Core i7-8550U CPU (4 cores).
  ○ Disk space: 30 GB minimum. The total disk space for eIF4F.analysis folder is 26.2 GB, including all downloads and the analysis results.

## Step-by-step method details

The package includes one initiation step, and seven major analysis steps. User can execute each analysis step with one exported function that calls a group of internal functions for data process, data analysis and plotting to achieve a set of analyses. This package organizes all functions related to each analysis step together as one R script under ∼/eIF4F.analysis/R (file names in bold, Figure 2E). Although data process, analysis and result plotting functions are set as internal functions, users can access their source code from R scripts and modify the input parameter outside this package for their own usages. The following section explains the definition and organization of each function. The detailed documentation of all (exported and internal) functions within the package is available at the following weblink: https://a3609640.github.io/eIF4F.analysis/reference/index.html.

Analysis.R contains ten exported functions in the package to initialize package and to execute all analyses presented in Wu and Wagner (2021).^1^ Users can simply execute the following command in RStudio to get all analyses performed and results stored under ∼/eIF4F.analysis/eIF4F_output.

> source("∼/eIF4F.analysis/Script/Analysis.R")

### Step-1: Library initialization

**Timing: < 5 min**

Initialization processes rely on three exported functions to define subdirectories for output data, to define the graphic formats (font size and style), and to load data from downloaded data files. The definitions of three initialization functions were stored in ∼/eIF4F.analysis/R/Load.R.

1. Run the following command line to create the output directories to store the output files.

> initialize_dir()

***Note:***initialize_dir() creates sub-directories under ∼/eIF4F.analysis/eIF4F_output (Figure 2F) to store the output results for each analysis step.

2. Run the following command line to create variables that define font type, size, and orientation for plotting.

> initialize_format()

**Figure 3:**
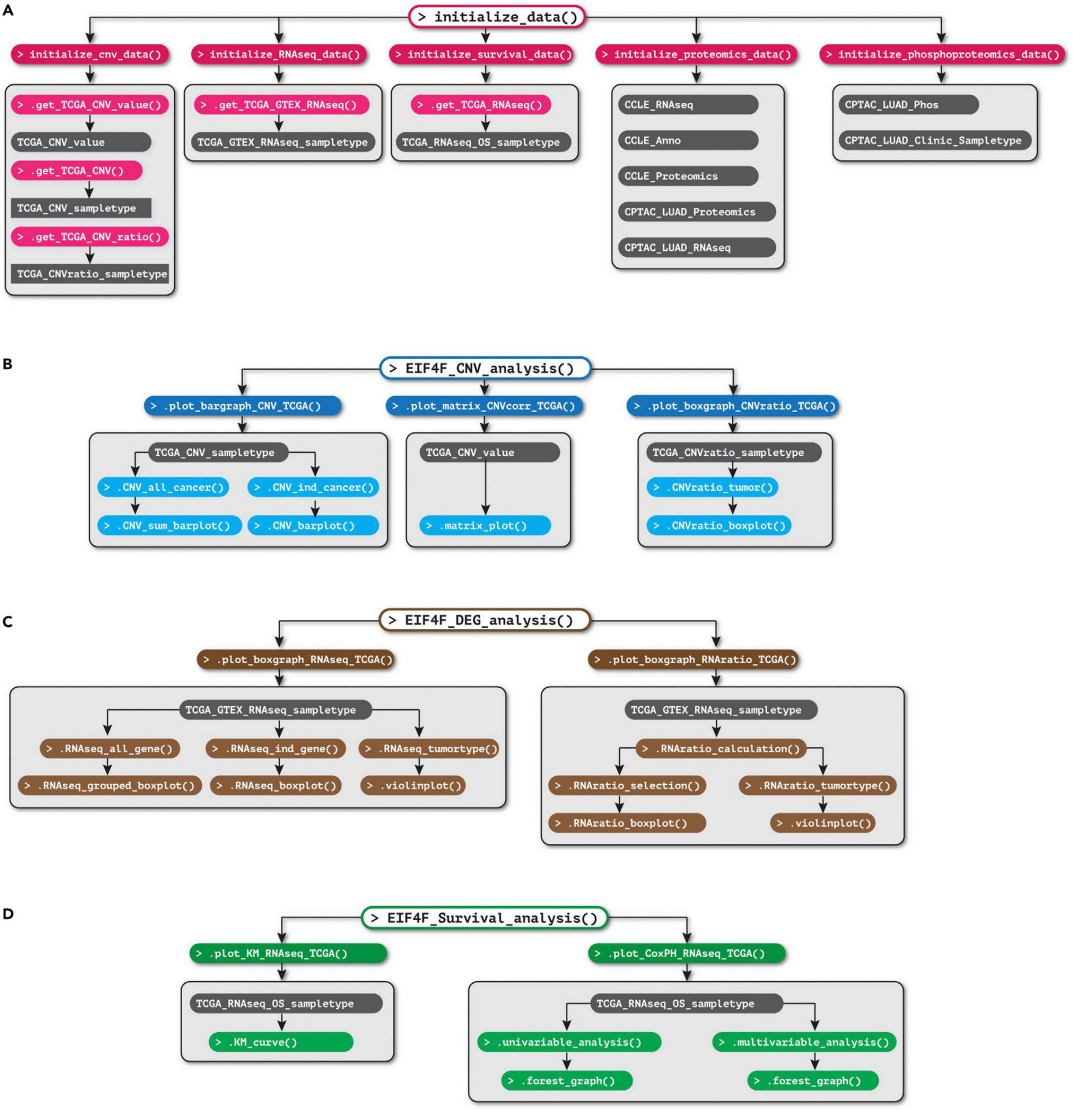
Illustration of internal code structure for analysis functions (A) The internal code structure for initialize_data(). (B) The internal code structure for EIF4F_CNV_analysis(). (C) The internal code structure for EIF4F_DEG_analysis(). (D) The internal code structure for EIF4F_Survival_analysis().

3. Run the following command line to acquire omics datasets from the download data files.
  > initialize_data()
  ***Note:***initialize_data() is a wrapper of five data initialization functions that import, trim, clean up and merge datasets from the download data files. These data initialization functions have side effects that populate twelve global variables that process and merge omics and annotation data by sample IDs for the following analysis steps. The data contained by these global variables are available in the form of data frames, as input for our analysis functions, but do not show on the user’s workspace. For users to access them, the data initialization step saves twelve global variables as csv files under ∼/eIF4F.analysis/eIF4F_output/ProcessedData folder (Figure 2G).
  ***Alternatives:*** Five individual data initialization functions are also accessible to users for execution. Instead of running the wrapper function initialize_data() to generate all twelve global variables at once, users can generate global variables related to each analysis function with the following individual data initialization functions.
  > initialize_cnv_data()
  > initialize_RNAseq_data()
  > initialize_survival_data()
  > initialize_proteomics_data()
  > initialize_phosphoproteomics_data()
  a. initialize_cnv_data() reads all CNV related datasets from TCGA, with a few internal functions. The implementation details of each operation are in ∼/eIF4F.analysis/R/CNV.R. initialize_cnv_data() sets the values of three global variables TCGA_CNV_value, TCGA_CNV_sampletype and TCGA_CNVratio_sampletype for CNV analysis (step-2) and stores them as “TCGA_CNV_value.csv”, “TCGA_CNV_sampletype.csv” and “TCGA_CNVratio_sampletype.csv” under the ProcessedData folder.
    i. TCGA_CNV_value contains the unthresholded CNV value data of tumors from all TCGA cancer types combined, and comes from the dataset, "Gistic2_CopyNumber_Gistic2_all_data_by_genes".
    ii. TCGA_CNV_sampletype contains the threshold CNV dataset of tumors from all combined TCGA cancer types. It comes from two datasets: "Gistic2_CopyNumber_Gistic2_all_thresholded.by_genes", and the annotation dataset "TCGA_phenotype_denseDataOnlyDownload.tsv".
    iii. TCGA_CNVratio_sampletype contains the data of CNV ratios in tumors vs. adjacent normal tissues from individual TCGA cancer types. It comes from two datasets: "broad.mit.edu_PANCAN_Genome_Wide_SNP_6_whitelisted.gene.xena", and the annotation dataset, "TCGA_phenotype_denseDataOnlyDownload.tsv".
  b. initialize_RNAseq_data() reads the recomputed RNAseq data from both TCGA and GTEx. The implementation details of each operation are within the ∼/eIF4F.analysis/R/DEG.R file. initialize_RNAseq_data() sets one global variable TCGA_GTEX_RNAseq_sampletype for the gene expression analysis (step-3), PCA (step-5) and correlating gene analysis (step-6), and stores TCGA_GTEX_RNAseq_sampletype as “TCGA_GTEX_RNAseq_sampletype.csv” in the ProcessedData folder.
    i. TCGA_GTEX_RNAseq_sampletype comes from the recomputed RNAseq dataset from both TCGA and GTEx, "TcgaTargetGtex_RSEM_Hugo_norm_count", and the annotation dataset "TcgaTargetGTEX_phenotype.txt".
  c. initialize_survival_data() reads the RNAseq and patient survival data from TCGA. The implementation details of this operation are within the ∼/eIF4F.analysis/R/Survival.R file. This function sets the global variable TCGA_RNAseq_OS_sampletype for survival analysis (step-4), and store TCGA_RNAseq_OS_sampletype as “TCGA_RNAseq_OS_sampletype.csv” inside the ProcessedData folder.
    i. TCGA_RNAseq_OS_sampletype comes from three datasets: the RNAseq dataset "EB++AdjustPANCAN_IlluminaHiSeq_RNASeqV2.geneExp.xena", the survival dataset "Survival_SupplementalTable_S1_20171025_xena_sp" and the annotation dataset "TCGA_phenotype_denseDataOnlyDownload.tsv".
  d. initialize_proteomics_data() reads the proteomics related data from CCLE and CPTAC LUAD, including: proteomics data, annotation data for cancer types, RNAseq data for the correlation analysis on protein RNA levels (step-7 and step-8). The implementation details of this operation are within the ∼/eIF4F.analysis/R/RNAProCorr.R file.
    i. This function sets three global variables for the CCLE data: (1) CCLE_RNAseq contains the RNAseq data derived from "CCLE_expression_full.csv", (2) CCLE_Anno contains the annotation data derived from "sample_info.csv", and (3) CCLE_Proteomics contains the protein expression level data derived from "protein_quant_current_normalized.csv". This function stores CCLE_RNAseq, CCLE_Anno and CCLE_Proteomics as “CCLE_RNAseq.csv”, “CCLE_Anno.csv”, and “CCLE_Proteomics.csv” inside the ProcessedData folder.
    ii. This function sets two global variables as data frames for the CPTAC LUAD data published in Gillette et al. (2020)^7^: (1) CPTAC_LUAD_Proteomics contains proteomics data from "Protein.xlsx", and (2) CPTAC_LUAD_RNAseq contains RNAseq data from "RNA.xlsx". This function stores CPTAC_LUAD_Proteomics and CPTAC_LUAD_RNAseq as “CPTAC_LUAD_Proteomics.csv” and “CPTAC_LUAD_RNAseq.csv” files in the ProcessedData folder.
  e. initialize_phosphoproteomics_data() reads phospho-proteomics-related data from CPTAC LUAD, and sets two global variables with data frames for the protein expression analysis (step-8). The implementation details of this operation are in the ∼/eIF4F.analysis/R/Proteomics.R file. This function stores two global variables CPTAC_LUAD_Phos and CPTAC_LUAD_Clinic_Sampletype as “CPTAC_LUAD_Phos.csv” and “CPTAC_LUAD_Clinic_Sampletype.csv” in the ProcessedData folder.
    i. CPTAC_LUAD_Phos contains the phosphoproteomics data published in Gillette et al. (2020)^7^ as "Phos.xlsx".
    ii. CPTAC_LUAD_Clinic_Sampletype contains the annotation data and is derived from "S046_BI_CPTAC3_LUAD_Discovery_Cohort_Clinical_Data_r1_May2019.xlsx" and "S046_BI_CPTAC3_LUAD_Discovery_Cohort_Samples_r1_May2019.xlsx".
      **CRITICAL:** The first run of data initialization functions creates and stores the processed data from download files. Following runs of the initialization functions will check the existence of processed data files and read them, which take a much shorter time to complete than the first run. Figure 3A summarizes the internal code structure for five data initialization functions, and shows twelve global variables in dark gray boxes.

### Step-2: Analyze the copy number variation (CNV) status of *EIF4F* genes

**Timing: < 1 min**

This step performs three types of analyses on CNV statuses of *EIF4F* genes across TCGA tumors and creates the analysis results both on screen and as pdf files stored in ∼/eIF4F.analysis/eIF4F_output/CNV folder.

4. Run the following command line in RStudio.

**Figure 4:**
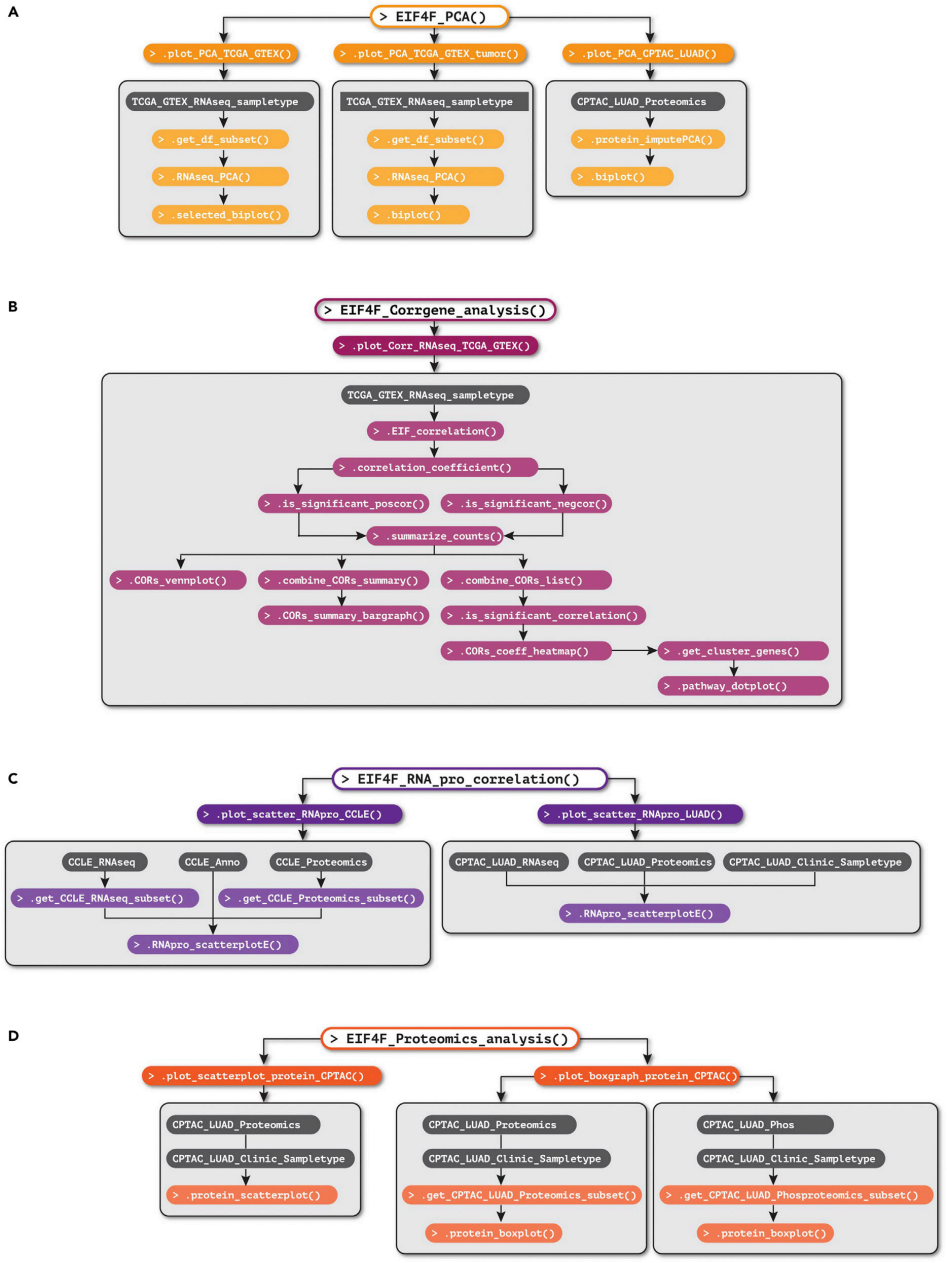
Illustrations of internal code structures for analysis functions (A) The internal code structure for EIF4F_PCA(). (B) The internal code structure for EIF4F_Corrgene_analysis(). (C) The internal code structure for EIF4F_RNA_pro_correlation(). (D) The internal code structure for EIF4F_Proteomics_analysis().

**Figure 5:**
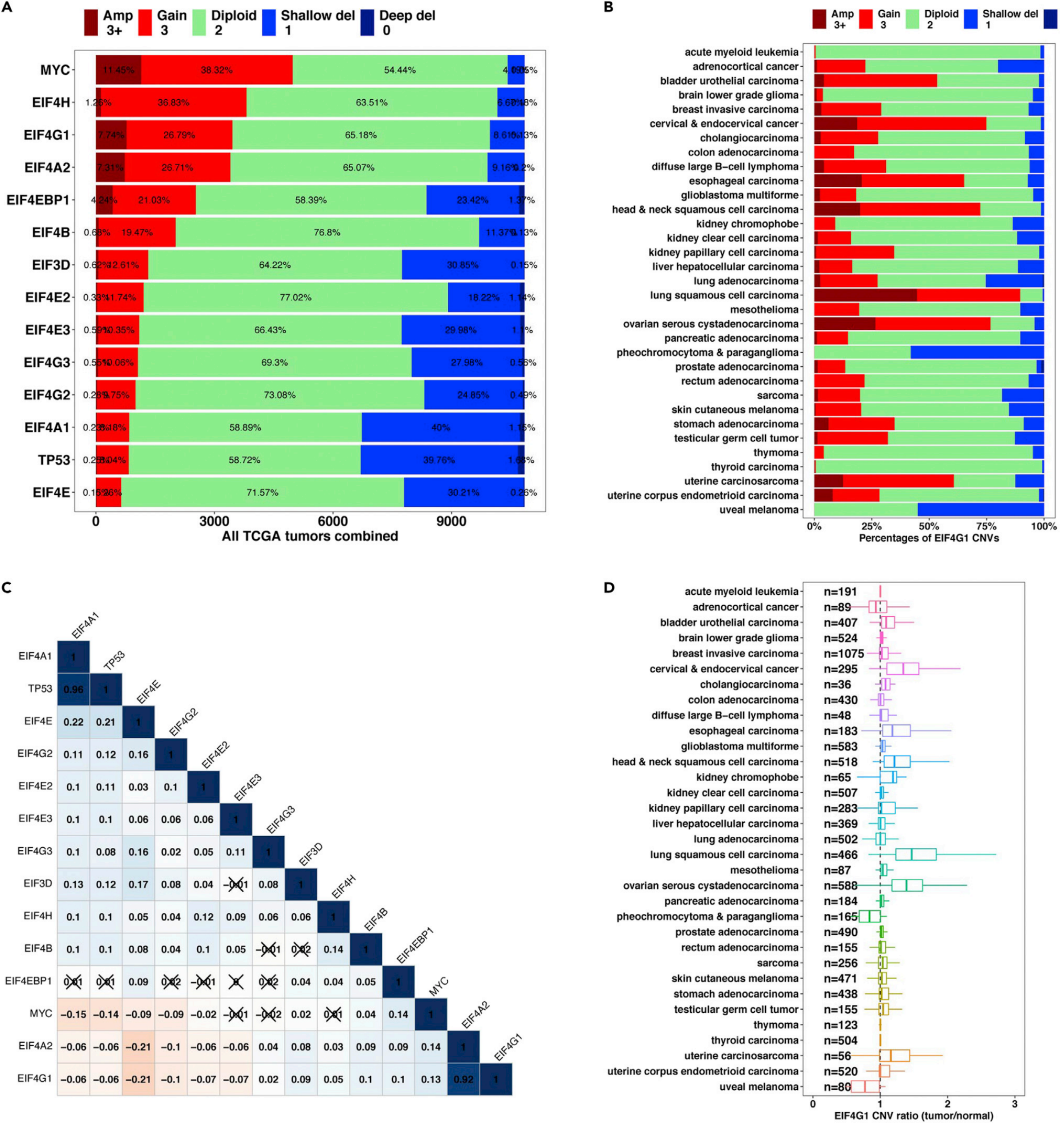
Example CNV analysis outputs from EIF4F_CNV_analysis() (A) The stacked bar plot shows the overall CNV statuses for EIF4F genes in all tumors combined from 33 TCGA cancer types. Percentage contributions of each group are labeled on the bars. (B) The stacked bar plot shows the CNV status for a single EIF4F gene in individual TCGA cancer types. (C) The matrix plot shows the co-occurrence of the EIF4F CNV statuses. Each cell is labeled with the Pearson correlation coefficient, and ‘‘X’’ indicate statistical insignificance (p > 0.05, p values not shown). (D) The boxplots show, across 33 TCGA cancer types, ratios of EIF4G1 CNV values in malignant tumors to its average CNV value in normal adjacent tissues (NATs) of the same cancer type. Adapted from Wu and Wagner (2021).^1^

> EIF4F_CNV_analysis()

***Note:***EIF4F_CNV_analysis() is a wrapper function of three internal composite functions that take input data frames and call internal functions for analysis. Figure 3B summarizes the internal code structure for this step. The detailed definitions of all internal functions are in ∼/eIF4F.analysis/R/CNV.R.

***Note:***.plot_bargraph_CNV_TCGA() takes the data frame TCGA_CNV_sampletype and calculates the frequency of each CNV status for *EIF4F* genes in all tumors combined from 33 TCGA cancer types. Its output is a stacked bar plot that ranks the *EIF4F* gene by the frequencies of copy number gain (Figure 5A). .plot_bargraph_CNV_TCGA() also calculates the frequency of CNV status from individual TCGA cancer types. Its output is a number of stacked bar plots, in which cancer types are listed in the alphabetical order (e.g., Figure 5B).

***Note:***.plot_matrix_CNVcorr_TCGA() takes the data frame TCGA.CNV.value and generates a correlation matrix with the unthresholded CNV value data of tumors from all TCGA cancer type combined. This function calculates the correlation coefficients between all pairs of EIF4F genes, and plots the correlation matrix (Figure 5C), using the cor.mtest() and corrplot() functions from “corrplot” package.

***Note:***.plot_boxgraph_CNVratio_TCGA() takes the data frame TCGA_CNVratio_sampletype and generates boxplots for CNV ratios in tumors vs. normal adjacent tissues (NATs) from individual TCGA cancer types. This function produces a CNV ratio boxplot for each gene in individual TCGA cancer types (e.g., Figure 5D).

### Step-3: Compare the gene expression and ratio of *EIF4F* genes

**Timing: < 1 min**

This step compares the *EIF4F* RNA abundances and ratios across TCGA tumors, and saves the results in the ∼/eIF4F.analysis/eIF4F_output/DEG folder. In this step, means of log-transformed gene expression values or RNA ratios are calculated in different samples, and compared by t-test.

5. Run the following command line in RStudio.

**Figure 6:**
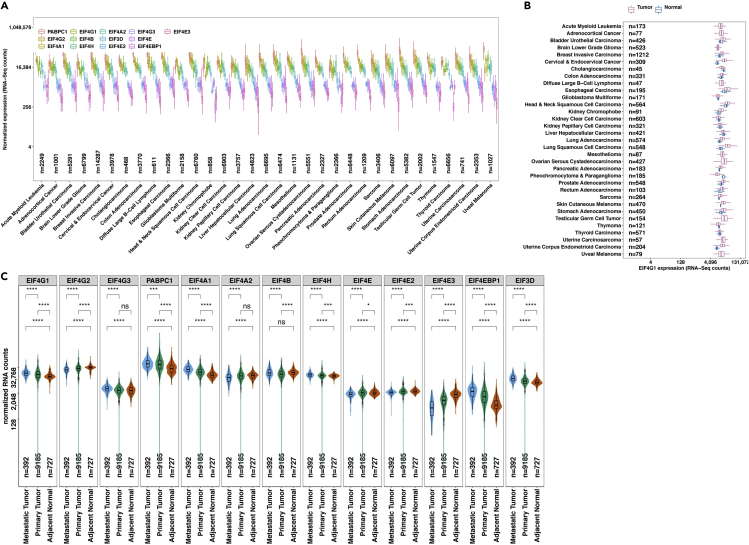
Example expression analysis outputs from EIF4F_DEG_analysis() (A) The boxplot represents the comparison of RNAseq expressions across different cancer types. (B) The box and whisker plot shows corresponding mRNA expression of the same gene in tumor samples and normal adjacent tissues (NATs). (C) The violin plots show the indicated mRNA expression (transcripts per million), in all TCGA cancer patient samples combined. The two-tailed Student’s t tests were performed. ns, not significant; ∗P ≤ 0.05; ∗∗p ≤ 0.01; ∗∗∗p ≤ 0.001; ∗∗∗∗p ≤ 0.0001. Adapted from Wu and Wagner (2021).^1^

**Figure 7:**
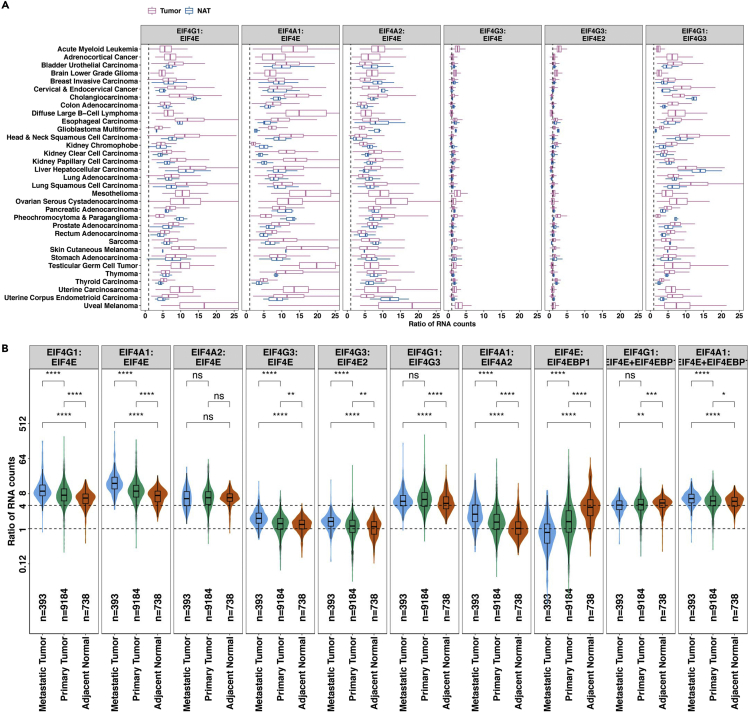
Example gene expression ratio analysis outputs from EIF4F_DEG_analysis() (A) The boxplots depict ratios of RNA counts between two genes, computed from each sample of a particular cancer type, for tumors and NATs considered separately. (B) The violin plots compare RNA ratios within metastatic tumors, primary tumors, or NATs in all 33 TCGA cancer study groups combined. Dashed lines mark the ratios of 1:1 and 4:1 in all panels. The two-tailed Student’s t tests were performed. ns, not significant; ∗p ≤ 0.05; ∗∗p ≤ 0.01; ∗∗∗p ≤ 0.001; ∗∗∗∗p ≤ 0.0001. Adapted from Wu and Wagner (2021).^1^

> EIF4F_DEG_analysis()

***Note:***EIF4F_DEG_analysis() is a wrapper function of two internal composite functions that take input data frames and call internal functions for analysis. Figure 3C summarizes the internal code structure for this step. The detailed definitions of all internal functions are in ∼/eIF4F.analysis/R/DEG.R.

***Note:***.plot_boxgraph_RNAseq_TCGA() takes the data frame TCGA_GTEX_RNAseq_sampletype and performs three analyses on RNAseq data. It compares the RNA abundance of all *EIF4F* genes in tumors from 33 TCGA cancer types in a box plot (Figure 6A). Then it compares the expression of each *EIF4F* gene in tumors vs. NATs (e.g., Figure 6B). Finally, it compares the RNA expression in primary, metastatic tumors vs. NATs from all combined TCGA cancer types to produce violin plots (Figure 6C).

***Note:***.plot_boxgraph_RNAratio_TCGA() takes the data frame TCGA_GTEX_RNAseq_sampletype to calculate the RNA ratios of input genes within each TCGA sample, and performs two analyses on the RNA ratios. It first compares RNA ratio in tumors vs. NATs from individual TCGA cancer types by boxplots (e.g., Figure 7A). It then compares RNA ratios in primary, metastatic tumors vs. NATs from all combined TCGA cancer types to produce violin plots (Figure 7B).

### Step-4: Correlate *EIF4F* gene expression to patient survival probability

**Timing: < 1 min**

This step performs two types of survival analyses on *EIF4F* gene expression in TCGA tumors, and output results on screen and to the ∼/eIF4F.analysis/eIF4F_output/Survival folder.

6. Run the following command line in RStudio.

**Figure 8:**
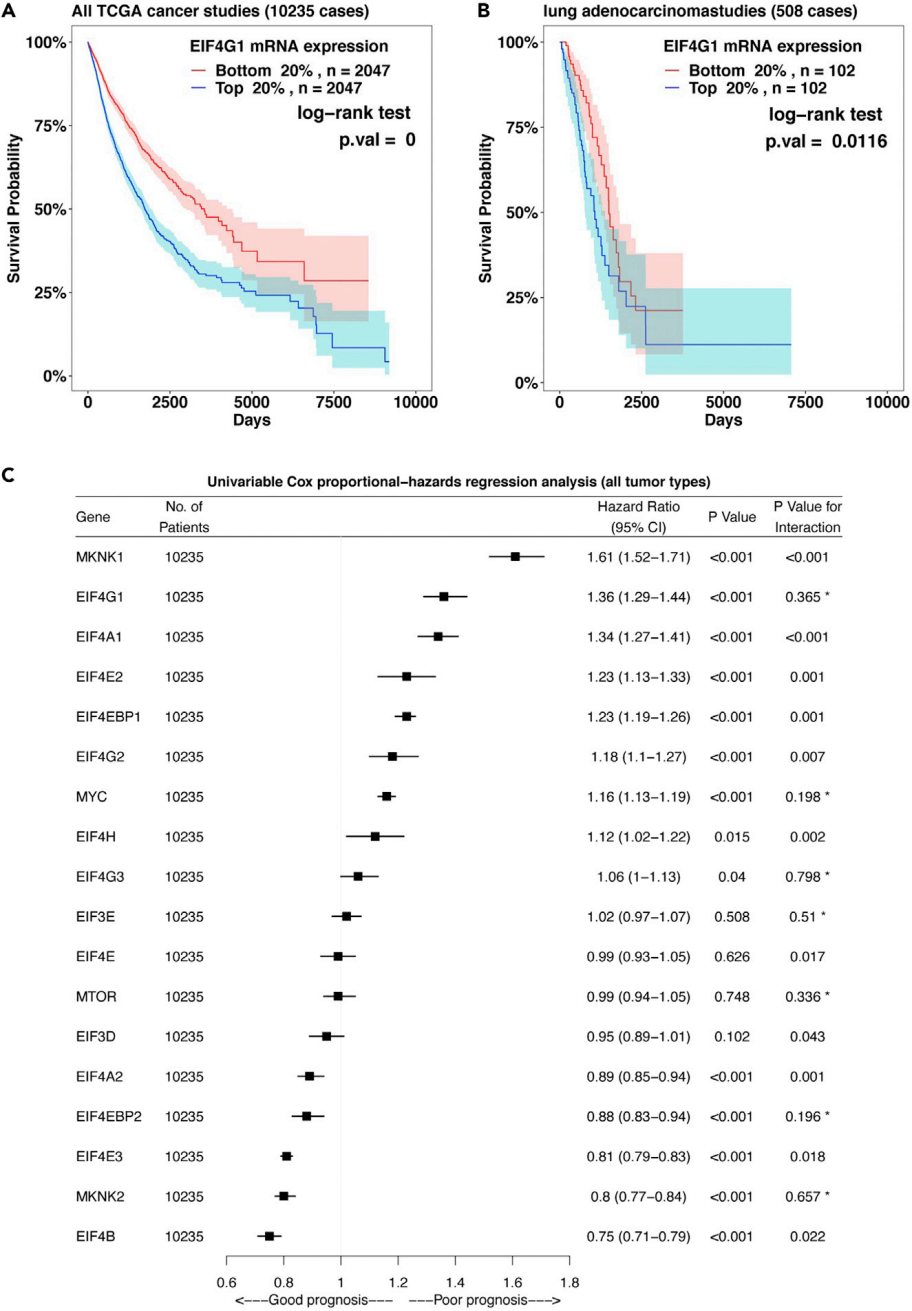
Example survival analysis outputs from EIF4F_Survival_analysis() (A) The KM plot of survival probabilities of TCGA patients with cancer according to mRNA expressions of *EIF4G1* in their tumors. Two groups of patients with the top or bottom 20% of gene expression in their tumors were selected from all 10,235 TCGA patients with cancer. Statistical significance of differences in survival probabilities between the two groups was determined by p values yielded from log-rank tests. The shaded areas around each curve depict a 95% confidence region for that curve. (B) The KM plot of survival probabilities of TCGA patients with lung adenocarcinoma according to mRNA expressions of *EIF4G1* in their tumors. Two groups of patients with the top or bottom 20% of gene expression in their tumors were selected from 508 TCGA patients with lung adenocarcinoma. (C) Univariable Cox proportional-hazards regression models for expression of translation initiation genes in all 10,235 patients with cancer from TCGA. The p value indicates the statistical significance of association between gene expression and survival (i.e., a significant fit). Adapted from Wu and Wagner (2021).^1^

> EIF4F_Survival_analysis()

***Note:***EIF4F_Survival_analysis() is a wrapper function to call two internal composite functions that take input data frames and call internal functions for analysis. Figure 3D summarizes the internal code structure for this step. The detailed definitions of all internal functions are in ∼/eIF4F.analysis/R/Survival.R.

***Note:***.plot_KM_RNAseq_TCGA() takes the data frame TCGA_RNAseq_OS_sampletype and performs Kaplan-Meier (KM) analysis to associate survival probabilities with gene expression. This function takes arbitrary gene expression cutoff, 0.2 for 20% or 0.3 for 30%, to stratify the patient groups based on the top or bottom precents of gene expression within their tumors. This function performs KM analysis on all combined TCGA cancer types (e.g., Figure 8A) or individual cancer type such as “lung adenocarcinoma” (e.g., Figure 8B). This function imports the survfit() and survdiff() functions from the “survival” package to analyze the survival probabilities of patient groups, and produces the KM curve plots.

***Note:***.plot_CoxPH_RNAseq_TCGA() takes the data frame TCGA_RNAseq_OS_sampletype, and quantitatively relates patient survival and gene expression in tumors by the Cox proportional-hazards (PH) regression method. This function can perform survival analyses on all combined TCGA cancer types or individual cancer type such as “lung adenocarcinoma” as an argument. The function performs both univariable Cox-PH analysis using a single gene expression as the dependent variable (e.g., Figure 8C), and multivariable Cox-PH analysis to model patient survival and expressions of all initiation factors together. This composite function imports the analyse_multivariate() function from the “survivalAnalysis” package for regression model. Proportional hazard assumptions of Cox Regression are tested by coxph() and cox.zph() functions from the “survival” package. The resulting plots are produced with the forestplot() function in the “forestplot” package.

### Step-5: Principal component analysis on co-variation of *EIF4F* expression

**Timing: < 1 min**

This step performs the principal component analyses (PCA) on *EIF4F* expression in GTEx healthy tissues, or/and TCGA tumors, and output results to the ∼/eIF4F.analysis/eIF4F_output/PCA folder.

7. Run the following command line in RStudio.

**Figure 9:**
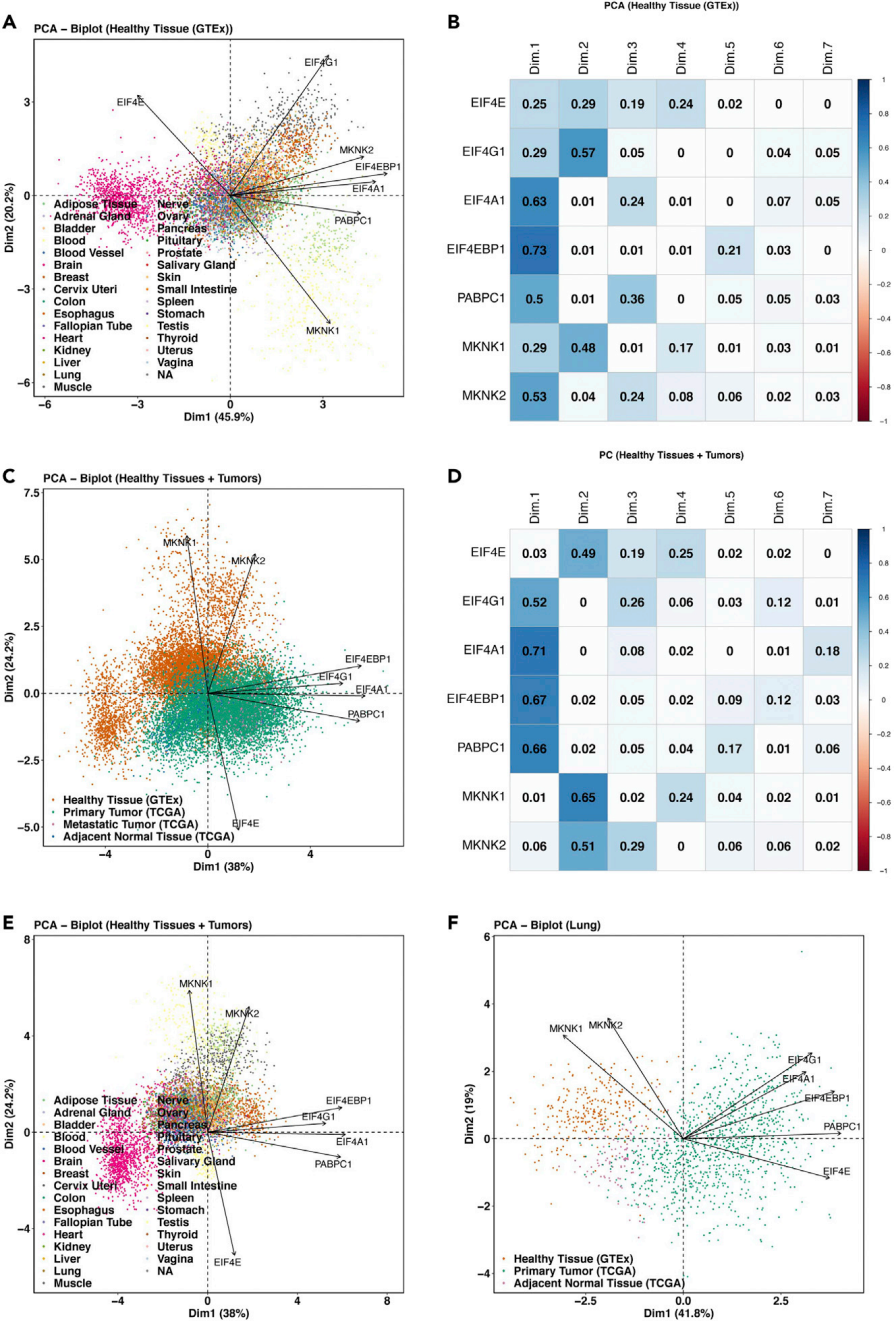
Example principal component analysis outputs from EIF4F_PCA() (A) PCA of RNA-seq-derived counts of *EIF4G1*, *EIF4A1*, *EIF4E*, *EIF4EBP1*, *PABPC1*, *MKNK1*, and *MKNK2* from 7,388 tissue samples of various healthy tissue types in GTEx. Tissue types were observations, colored for visualization but not used to construct PCs. (B) The matrix plot shows the cos2 value for the contribution of each gene to each PC, from the PCA of 7,388 tissue samples of various healthy tissue types in (A). (C) PCA of normalized RNA-seq-derived counts of indicated genes from 9,162 primary and 392 metastatic tumors from TCGA and 7,388 healthy tissue samples from GTEx. Sample types were colored after analysis, for visualization. (D) The matrix plot shows the cos2 value for the contribution of each gene to each PC, from the PCA of TCGA tumor samples and GTEx normal tissue samples in (C). (E) Healthy tissue selection from (C). The tissue types were not used as variables to construct PCs, but were colored afterwards for visualization. (F) PCA of standardized RNA-seq-derived counts of indicated genes from 1,011 primary lung tumors (517 LUADs and 494 LUSCs) and 109 NATs from TCGA LUAD and LUSC study groups, and 287 healthy lung tissues from GTEx. Sample types were colored after analysis, for visualization. Adapted from Wu and Wagner (2021).^1^

> EIF4F_PCA()

***Note:***EIF4F_PCA() is a wrapper function of two internal composite functions that take input data frames and call internal functions for analysis. Figure 4A summarizes the internal code structure for this step. The detailed definitions of all internal functions are in ∼/eIF4F.analysis/R/PCA.R.

***Note:***.plot_PCA_TCGA_GTEX() takes the data frame TCGA_GTEX_RNAseq_sampletype and selects RNAseq data of three sample types: TCGA primary tumors, TCGA metastatic tumors, or GTEx healthy tissues. It performs separate PCA on *EIF4F* expression within the selected sample types and produces PCA results as biplots (e.g., Figure 9A), scree and matrix plots (e.g., Figure 9B). This function call also performs PCA on combined samples of all TCGA primary tumors, TCGA metastatic tumors and GTEx healthy tissues, which produces biplots (e.g., Figure 9C), scree and matrix plots (e.g., Figure 9D), as well as subset biplots that label only cancer types or healthy tissue types (e.g., Figure 9E). This function imports PCA() function from the “FactoMineR” package for PCA, and fviz_pca_biplot(), fviz_eig(), get_pca_var() functions from the “factoextra” packages to produce biplots, scree and matrix plots.

***Note:***.plot_PCA_TCGA_GTEX_tumor() takes the data frame TCGA_GTEX_RNAseq_sampletype and selects RNAseq data from one specific TCGA cancer type and its matched healthy tissue from GTEx. This function performs PCA on *EIF4F* expression within the selected sample types and generates biplots (e.g., Figure 9F), scree and matrix plots for PCA results.

***Note:***.plot_PCA_CPTAC_LUAD() takes the data frame CPTAC_LUAD_Proteomics and selects proteomics data of input gene list. Due to the missing proteomics data for some inquired initiation proteins in the dataset, this function performs imputed PCA with the estim_ncpPCA() function from the “missMDA” package.

### Step-6: Analyze the correlating genes (CORs) of *EIF4F* subunits

**Timing: < 30 min**

This step analyzes the correlating genes of each *EIF4F* subunits from tumor or healthy samples, and outputs results to the ∼/eIF4F.analysis/eIF4F_output/COR folder.

8. Run the following command line in RStudio.

**Figure 10:**
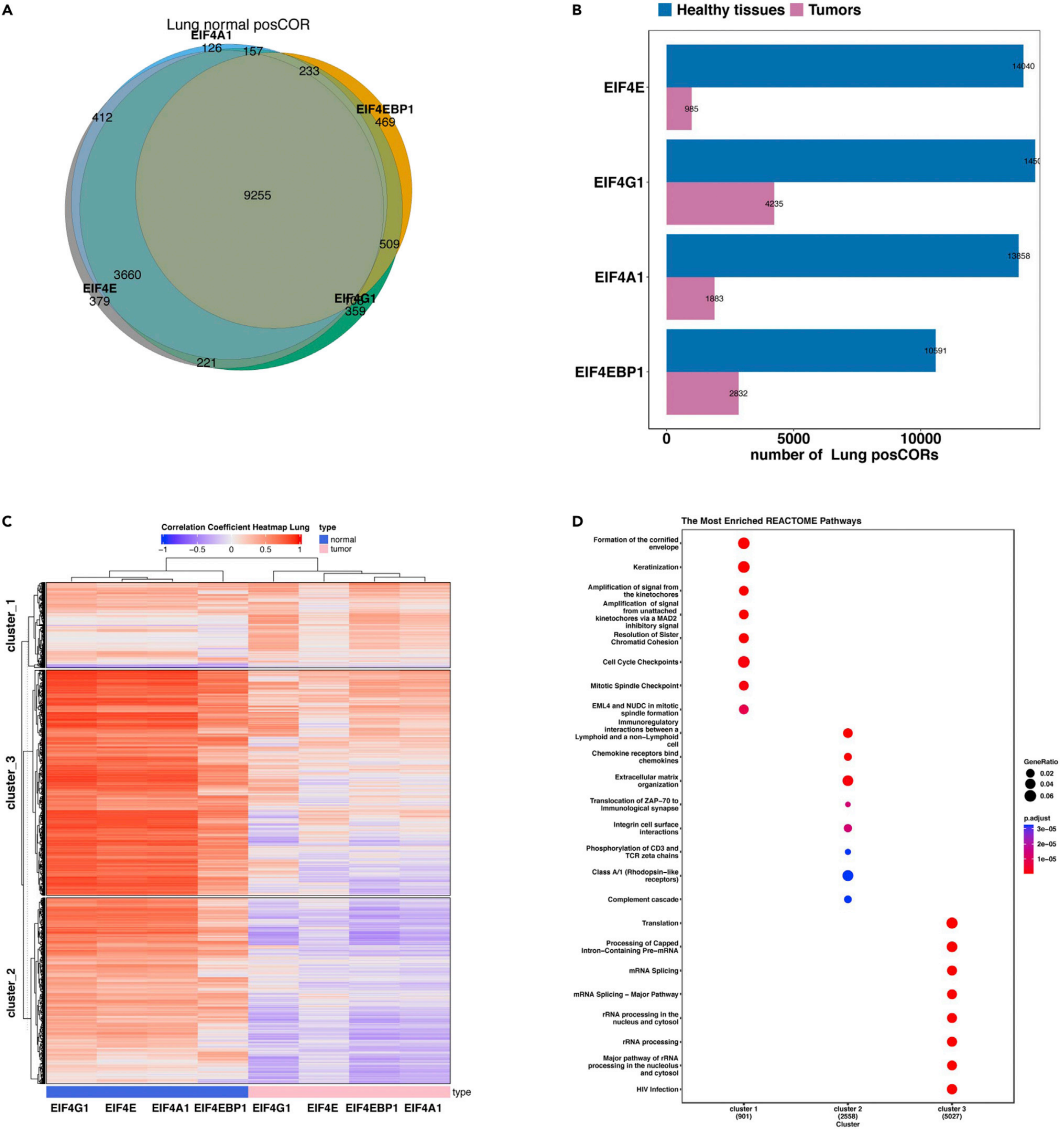
Example correlation gene analysis outputs from EIF4F_Corrgene_analysis() (A) Pearson’s correlation coefficients between *EIF4F* (*EIF4E*, *EIF4G1*, *EIF4A1*, or *EIF4EBP1*) and 58,582 other genes were calculated separately across 1,122 lung tumors from LUSC and LUAD TCGA study groups, or across 287 healthy lung tissues from GTEx. Genes with significant positive (R > 0.3) or negative (R < -0.3) correlations were selected for analysis. The Venn diagrams show overlapping posCOR counts for EIF4F genes in healthy lungs. (B) The bar plots show the numbers of posCORs identified for each EIF4F gene in tumors or in healthy tissues. (C) The heatmap shows the correlation strengths of posCORs and negCORs for *EIF4E*, *EIF4G1*, *EIF4A1*, and *EIF4EBP1* in healthy lungs and lung tumors. (D) The dot plot shows the enriched pathways in three clusters (K-means) of the heatmap (C) yielded by REACTOME pathway analysis. Adapted from Wu and Wagner (2021).^1^

> EIF4F_Corrgene_analysis()

***Note:***EIF4F_Corrgene_analysis() is a wrapper function to call one internal composite function that takes input data frames and calls internal functions for analysis. Figure 4B summarizes the internal code structure for this step. The detailed definitions of all internal functions are in ∼/eIF4F.analysis/R/COR.R.

***Note:***.plot_Corr_RNAseq_TCGA_GTEX() takes the data frameTCGA_GTEX_RNAseq_sampletype and selects RNAseq data from two sample types: TCGA tumors, or GTEx healthy tissues. This function separately identifies correlating genes (CORs) of *EIF4E*, *EIF4A1*, *EIF4G1*, and *EIF4EBP1* from tumor samples or from healthy tissues. The significant CORs for each *EIF4F* subunit are identified and classified as positive or negative CORs (posCORs and negCORs). This function analyzes the overlap posCORs or negCORs of four *EIF4F* subunits from tumor samples or healthy tissues as Venn plots (e.g., Figure 10A). This function counts the numbers of posCORs and negCORs, and plots them in bar graphs for comparison (e.g., Figure 10B).

***Note:***.plot_Corr_RNAseq_TCGA_GTEX() merges tumor and healthy correlation data, and clusters correlation strengths by similarity in heatmaps (e.g., Figure 10C), using the HeatmapAnnotation() and draw() functions from the “ComplexHeatmap” package. Then this function extracts the gene lists identified in the clustering analysis with the row_order() function from “ComplexHeatmap”. Finally, this function performs enriched pathway analysis on the extracted gene lists using the compareCluster() function from the “clusterProfiler” package, and produces dot plots (Figure 10D as an example).

**CRITICAL:** .plot_Corr_RNAseq_TCGA_GTEX() function only applies to *EIF4F* and related genes.

### Step-7: Analyze the correlation between RNA and protein expression

**Timing: < 1 min**

This step examines the RNA and protein expression correlation in CCLE and CPTAC lung adenocarcinoma (LUAD) datasets, and outputs results to the ∼/eIF4F.analysis/eIF4F_output/RNApro folder.

9. Run the following command line in RStudio.

**Figure 11:**
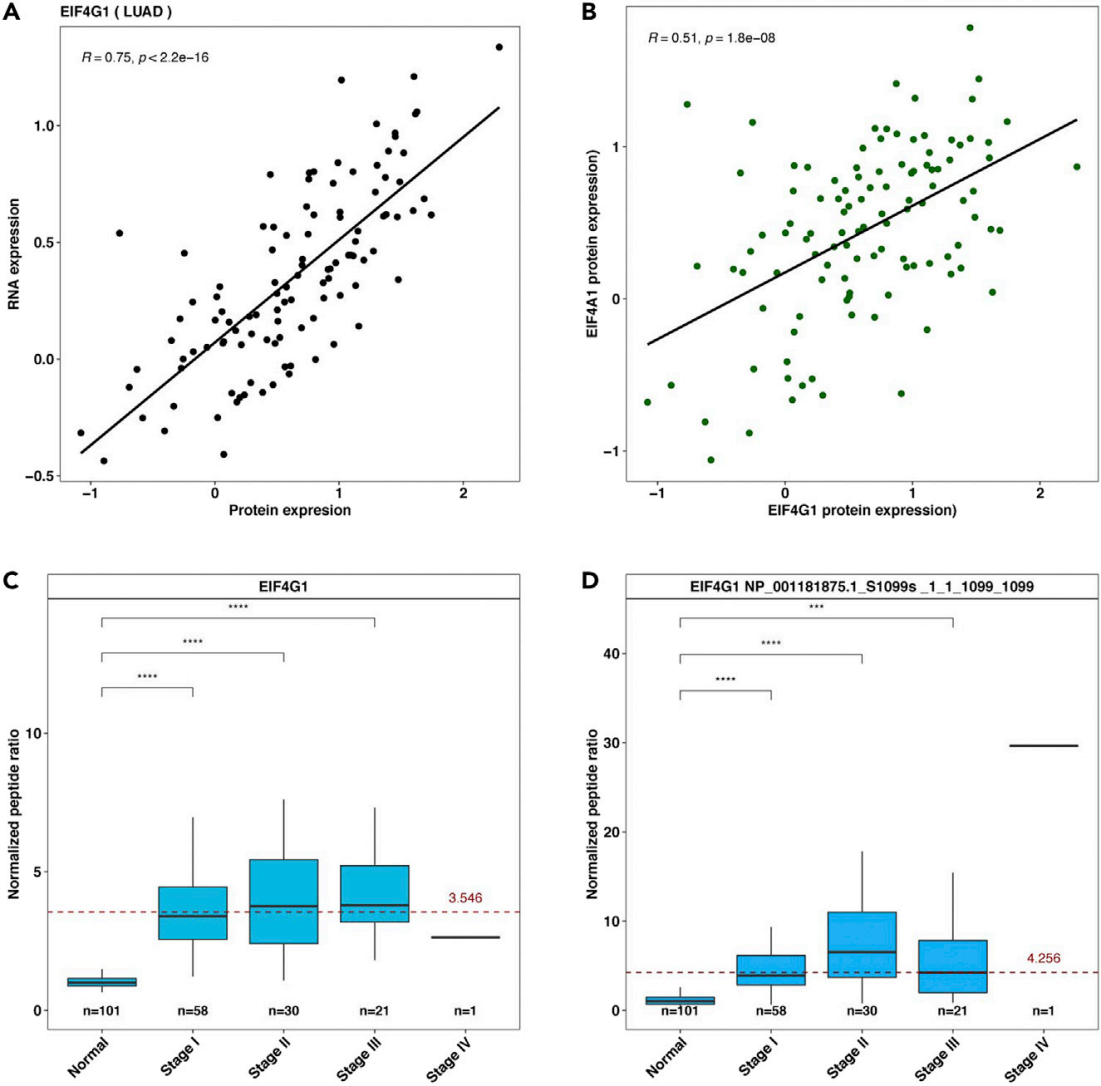
Example protein RNA correlation analysis outputs from EIF4F_RNA_pro_correlation() function, and example differential (phospho)protein expression analysis outputs from EIF4F_Proteomics_analysis() (A) The scatter plot shows the positive correlation between protein and mRNA expression levels of EIF4G1 across 109 LUADs from CPTAC. The significance of each correlation is indicated with a p value. (B) The scatter plot shows the correlation of eIF4G1 and eIF4A1 protein expression levels across 109 LUADs from CPTAC. The significance of each correlation is indicated with a p value. (C and D) The boxplots show, for eIF4G1, ratios to paired NATs (y axis) of mean peptide abundance. Depicted for NATs and LUADs at each tumor stage are: eIF4G1 whole protein (C) and phosphorylation at serine 1,099 (D). Mean expression from NATs was normalized at 1. The dashed red line marks average abundance in all tumor stages combined, relative to NATs. The two-tailed Student’s t tests were performed. ns, not significant; ∗p ≤ 0.05; ∗∗p ≤ 0.01; ∗∗∗p ≤ 0.001; ∗∗∗∗p ≤ 0.0001. Adapted from Wu and Wagner (2021).^1^

> EIF4F_RNA_pro_correlation()

***Note:***EIF4F_RNA_pro_correlation() is a wrapper function to call two internal composite functions that take input data frames and call internal functions for analysis. Figure 4C summarizes the internal code structure for this step. The detailed definitions of all internal functions are in ∼/eIF4F.analysis/R/RNAProCor.R.

***Note:***.plot_scatter_RNApro_CCLE() takes the data frames, CCLE_RNAseq and CCLE_Proteomics, and selects RNAseq and proteomics data for *EIF4F* genes. It performs the Pearson correlation analysis between proteomics and RNAseq levels, and produces the results as scatter plots.

***Note:***.plot_scatter_RNApro_LUAD() takes the data frames, CPTAC_LUAD_RNAseq and CPTAC_LUAD_Proteomics, and selects RNAseq and proteomics data for *EIF4F* genes. It performs the correlation analysis between the proteomics and RNAseq data for *EIF4F* genes and produces scatter plots (e.g., Figure 11A).

### Step-8: Analyze the protein co-expression and (phospho)protein expression across tumor stages

**Timing: < 5 min**

This step examines the protein co-expression and compares (phospho)protein expression in different tumor stages, and outputs results to the ∼/eIF4F.analysis/eIF4F_output/proteomics folder.

10. Run the following command line in RStudio.

> EIF4F_Proteomics_analysis()

***Note:***EIF4F_Proteomics_analysis() is a wrapper function to call two internal composite functions that take input data frames and call internal functions for analysis. Figure 4D summarizes the internal code structure for this step. The detailed definitions of all internal functions are in ∼/eIF4F.analysis/R/proteomics.R.

***Note:***.plot_scatterplot_protein_LUAD() takes the data frame CPTAC_LUAD_Proteomics and selects the proteomics data of tumor samples. It analyzes the correlation between two input proteins across the LUAD tumor samples (e.g., Figure 11B).

***Note:***.plot_boxgraph_protein_CPTAC() takes the data frames CPTAC_LUAD_Proteomics, CPTAC_LUAD_Phos and CPTAC_LUAD_Clinic_Sampletype, and selects the proteomics, phosphoproteomics and clinical stage data. It generates boxplots to compare the protein (e.g., Figure 11C) or phophosphorylation (e.g., Figure 11D) levels in tumors of different clinical stages.

## Expected outcomes

All analysis results are produced as plots on screen and as pdf files in output file directories. Examples of results from each analysis step are shown in Figures 5, 6, 7, 8, 9, 10, and 11. The complete analysis results will be saved as 338 files in 17 directories (Figure 2F).

The execution of this package relies on specific exported functions and does not allow parameter input within the package. However, for each analysis step, the internal composite functions, the required input data frames, and the dependent functions are stored within one R file (Figure 2E). Most internal composite functions allow changes of gene inputs. To perform any analyses with its own gene input, users can open the R file for each analysis step in ∼/eIF4F.analysis/R and find the relevant internal functions. Figures 3 and 4 explain the internal code structure, which employs internal functions as individual building blocks. Users can combine the internal functions provided by this R package to assemble their own pipeline in a new R project. The desired input data frames for each internal composite function can be accessed from ProcessedData folder in Figure 2G.

## Limitations

eIF4F.analysis package relies on many dependent packages for data analysis and plotting. Because those dependent packages are widely used for individual applications, their combined usage provides great convenience for users to achieve a thorough understand of eIF4F functions. However, a major limitation of this approach is that incompatibility may occur with future version changes, since those packages are maintained independently of each other.

This package aims to provide an easy method to reliably reproduce the results from Wu and Wagner (2021),^1^ and to allow users to access and understand the associated code (which has been refactored for clarity since the original publication). Due to the large number dependencies and analyses this package performs, the exported functions are not designed to contain input parameters for users. Instead, input parameters such as gene names or sample types have been hard coded in the internal functions.

## Troubleshooting

### Problem 1

Error messages during Preparation three: installing and loading eIF4F.analysis.

Attaching package: ‘eIF4F.analysis’

The following objects are masked _by_ ‘.GlobalEnv’:

initialize_data, initialize_dir, initialize_format

### Potential solution

It means that you have objects (functions, usually) present in your global environment with the same name as (exported) things in your package. Clean the environment and reload the package.

### Problem 2

While running EIF4F_Corrgene_analysis() at step-6: analyze the correlating genes (CORs) of EIF4F subunits, you run into the following error:

> EIF4F_Corrgene_analysis()

Failed with error: org.Hs.egPFAM is defunct. Please use select() if you need access to PFAM or PROSITE accessions.

***Note:*** The object org.Hs.egPFAM has not been not used for any operation in the script, thus this error message does not affect the results of the analyses.

### Potential solution

Run the following command to reload the “org.Hs.eg.db” package. If the same error message of defunct org.Hs.egPFAM appears, please reinstall the "org.Hs.eg.db" package to the latest version (3.15.0).

> library(org.Hs.eg.db)

## Resource availability

### Lead contact

Further information and requests for resources should be directed to and will be fulfilled by the lead contact, Gerhard Wagner (gerhard_wagner@hms.harvard.edu).

### Materials availability

This study did not generate new unique reagents.

## Data Availability

This paper analyzes existing, publicly available data. The source datasets are listed in the key resources table. All original code has been deposited at GitHub: https://github.com/a3609640/eIF4F.analysis and archived on Zenodo: https://doi.org/10.5281/zenodo.7226224. The documentations for this package are available at GitHub: https://a3609640.github.io/eIF4F.analysis. All original code is publicly available as of the date of publication. DOIs are listed in the key resources table. Any additional information required to reanalyze the data reported in this paper is available from the lead contact upon request.
